# *In vitro* evaluation of the enhancement of glass ionomer cement features by using chitosan and nanodiamond

**DOI:** 10.1590/0103-644020246215

**Published:** 2025-04-14

**Authors:** Murilo Henrique Torres da Silva, Henrico Badaoui Strazzi-Sahyon, Renata Gallo, Ticiane Cestari Fagundes, André Luiz Fraga Briso, Victoria Tamara Perchyonok, Paulo Henrique dos Santos

**Affiliations:** 1 Department of Preventive and Restorative Dentistry, Araçatuba School of Dentistry, São Paulo State University - UNESP, Araçatuba, SP, Brazil.; 2 Department of Dental Materials and Prosthodontics, Araçatuba School of Dentistry, São Paulo State University - UNESP, Araçatuba, SP, Brazil.; 3 Department of Prosthodontics and Periodontology, Bauru School of Dentistry, University of Sao Paulo - USP, Bauru, SP, Brazil.; 4 Division of Biomaterial and Biomedical Sciences, Department of Oral Rehabilitation and Biosciences, Oregon Health & Science University, OHSU, Portland, OR, USA.; 5 VTPCHEM PTY LTD, Department of Research and Innovations, Melbourne, Australia.; 6 Dental Research Institute - Restorative Dentistry, Faculty of Dentistry, University of Toronto, Toronto, ON, Canada.

**Keywords:** chitosan, glass ionomer cement, hardness, nanodiamond, surface properties

## Abstract

This study investigated the influence of chitosan and nanodiamond incorporation on the surface, optical, and mechanical properties of glass ionomer cement. Total 56 samples (5 mm diameter and 2 mm thickness) were prepared and divided into 4 groups according to the incorporation of chitosan and nanodiamond on Fuji II glass ionomer cement: Control group: no incorporation; 10%CH group: incorporation of 10% chitosan; 10%ND group: incorporation of 10% of nanodiamond; 5%CH-5%ND group: incorporation of 5% chitosan and 5% nanodiamond (n=14). Analyses of color stability, surface roughness, fluorescence intensity, microhardness, morphology, and chemical composition were investigated. Additionally, water sorption, hygroscopic expansion, contact angle, surface free energy, and total free energy of interaction were also assessed. After the initial readings, the samples were individually stored in red wine solution for 28 days. Data were subjected to ANOVA followed by Tukey´s test (α=.05). Aging in wine solution altered the optical, mechanical, and surface properties of glass ionomer cement regardless of the incorporation of the compound (P<.05). 10% chitosan-incorporated glass ionomer cement promoted higher color alteration, surface roughness, and water sorption after aging (P<.05). 10% nanodiamond-incorporated glass ionomer cement showed higher microhardness compared to the other groups before aging (P<.05), however there were no differences among them after aging (P>.05). In general, no differences between the 5% chitosan- and 5% nanodiamond-incorporated glass ionomer cement and control groups were noted on the evaluated analyses (P>.05). Thus, the incorporation of 5% chitosan and 5% nanodiamond is a satisfactory alternative for maintain the surface, optical, and mechanical properties of the glass ionomer cement.

## Resumo

Este estudo investigou a influência da incorporação de quitosana e nanodiamante nas propriedades de superfície, ópticas e mecânicas de um cimento de ionômero de vidro. Um total de 56 amostras (diâmetro de 5 mm e espessura de 2 mm) foram preparadas e divididas em 4 grupos experimentais de acordo com a incorporação de quitosana e nanodiamante no cimento de ionômero de vidro Fuji II: Grupo Controle: sem incorporação; Grupo 10%CH: incorporação de 10% de quitosana; Grupo 10%ND: incorporação de 10% de nanodiamante; Grupo 5%CH-5%ND: incorporação de 5% de quitosana e 5% de nanodiamante (n=14). Análises de estabilidade de cor, rugosidade de superfície, intensidade de fluorescência, microdureza, morfologia e composição química foram investigadas. Além disso, a sorção de água, expansão higroscópica, ângulo de contato, energia livre de superfície e energia livre total de interação também foram avaliados. Após as leituras iniciais, as amostras foram armazenadas individualmente em solução de vinho tinto por 28 dias. Os dados foram submetidos à ANOVA seguida pelo teste de Tukey (α=.05). O envelhecimento na solução de vinho alterou as propriedades ópticas, mecânicas e de superfície do cimento de ionômero de vidro independentemente da incorporação dos compostos (P<.05). O cimento de ionômero de vidro incorporado com 10% de quitosana promoveu maior alteração de cor, rugosidade de superfície e sorção de água após o envelhecimento (P<.05). O cimento de ionômero de vidro incorporado com 10% de nanodiamante apresentou maior microdureza em comparação com os outros grupos antes do envelhecimento (P<.05), no entanto, não houve diferenças entre eles após o envelhecimento (P>.05). Em geral, não foram observadas diferenças entre os grupos de cimento de ionômero de vidro incorporado com 5% de quitosana e 5% de nanodiamante e os grupos controle nas análises avaliadas (P>.05). Assim, a incorporação de 5% de quitosana e 5% de nanodiamante é uma alternativa satisfatória para manter as propriedades de superfície, ópticas e mecânicas do cimento de ionômero de vidro.

**Figure:**
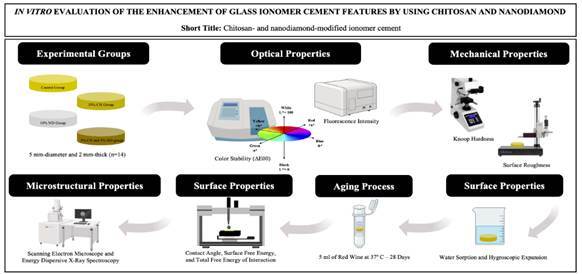

## Introduction

Introduced in the late 1960s by Wilson and Kent, glass ionomer cement is a tooth-colored material known for its unique properties ^1^ ^,^ ^2^ ^,^ ^3^ ^,^ ^4^. Its advancements, including chemical adhesion to tooth structures, tooth-like coefficient of thermal expansion, and biocompatibility, make it a satisfactory choice for atraumatic restorative treatment, temporary restoration, primary tooth restoration, and use in different techniques of restoration, and like luting or sealant agents ^1^ ^,^ ^2^ ^,^ ^3^ ^,^ ^4^. Furthermore, glass ionomer cement has anti-cariogenic action, as the substances present in their composition, when binding to the dental substrate, release fluoride ions over time ^5^.

Despite these advantages, ionomeric material used for atraumatic restorative treatment and permanent restorations have limitations such as low wear resistance and fragility over time, harming their clinical applicability, since they are vulnerable to tensions and forces originating from the stomatognathic system ^2^ ^,^ ^5^. Furthermore, the occurrence of bacterial plaque accumulation on surface materials could lead to unsatisfactory clinical conditions, such as unsuitable breath, secondary caries, bleeding, gingivitis, and in more severe cases periodontitis ^6^ ^,^ ^7^.

In this context, studies have investigated the incorporation of polysaccharides, such as chitosan, into restorative materials to enhance their clinical longevity in the oral cavity ^1^ ^,^ ^2^ ^,^ ^3^ ^,^ ^4^ ^,^ ^5^ ^,^ ^6^. Chitosan, a polysaccharide derived from chitin found in crustacean exoskeletons, is a biomaterial that has stood out in the incorporation of restorative materials due to its biocompatibility, biodegradability, adhesive properties to the dental substrate, anti-inflammatory and antibacterial properties, prevention of dental enamel demineralization, and inhibition of bacterial plaque accumulation, in addition to acting as a reinforcing material for the restorative compound ^5^ ^,^ ^8^. Although the exact mechanism of chitosan action is not well described in the literature, its incorporation has been widely used in different dental materials such as adhesive systems, mouthwashes, dentifrices, composite resins, and glass ionomer cement (1-5). Studies have demonstrated the effectiveness of the association of the chitosan substance with the antibacterial activity of *Streptococcus* present in the oral cavity, providing a satisfactory and beneficial performance, since these microorganisms are directly related to the development of caries ^1^ ^,^ ^8^

Due to the low wear resistance and fragility properties of conventional glass ionomer cement, it would be interesting to evaluate the incorporation of other biomaterials able to improve the mechanical properties of glass ionomer cement, including nanodiamond particles. Carbon-based nanomaterials, also called nanodiamonds, have been widely used in materials due to their properties of biocompatibility, hardness, thermal conductivity, and high mechanical strength ^9^ ^,^ ^10^ ^,^ ^11^. The reinforcement of materials by the incorporation of nanodiamond particles in polymeric matrices has shown interesting results ^9^ ^,^ ^10^ ^,^ ^11^. Thus, the evaluation of the chitosan and nanodiamond particles incorporation into glass ionomer cements would be promising, since they can provide satisfactory performance and longevity to the restorative material.

The current literature offers a limited number of *in-vitro* studies, with significant variability in the methods used to incorporate these compounds into glass ionomer cement ^1^ ^,^ ^2^ ^,^ ^3^ ^,^ ^4^. Most notably, the majority of research has focused on incorporating these compounds into the liquid phase rather than the powder phase ^1^ ^,^ ^2^ ^,^ ^3^. This underscores the need for further studies to thoroughly assess the impact of these compounds on the surface, optical, and mechanical properties of the material. Additionally, it highlights the importance of optimizing incorporation techniques, particularly in the powder phase, to achieve better outcomes in dental restorative applications.

In addition to the intrinsic properties of glass ionomer cements, some usual actions, such as the ingestion of food and drinks containing dyes can influence the structure of glass ionomer cements. The incorporation of dyes into the restorative material could occur due to the sorption capacity of the liquid by the organic matrix and the surface texture of the material that could act as an irregular structure resulting from the acidic action causing the chromatic change of the restoration ^12^. As a result of these reactions, staining becomes one of the main clinical concerns, leading to the replacement of restorations ^12^.

Ingestion of wine causes greater chances of staining due to the presence of dyes in its composition ^12^. In addition, the low pH is the crucial care factor of the interaction of this solution with the dental surfaces and restorative materials ^12^. The high concentration of ethanol and tannin can affect the surface integrity and cause the restoration change, in addition to allowing the softening of the organic matrix affecting the optical, mechanical, and surface properties of the glass ionomer cement ^12^.

Thus, this *in vitro* study aimed to determine the influence of chitosan and nanodiamond incorporation into a glass ionomer cement on the color stability, surface roughness, fluorescence intensity, microhardness, morphology, and chemical composition subjected to aging on red wine solution, as well as its effects on the water sorption, hygroscopic expansion, contact angle, surface free energy, and total free energy of interaction of the restorative material. The null hypotheses tested were: 1) Chitosan and nanodiamond incorporation would not influence the optical, mechanical, and surface properties of the glass ionomer cement; 2) aging in red wine solution would not cause changes in the properties of the restorative material.

## Materials and methods

### Specimen Preparation

**Figure 1:**
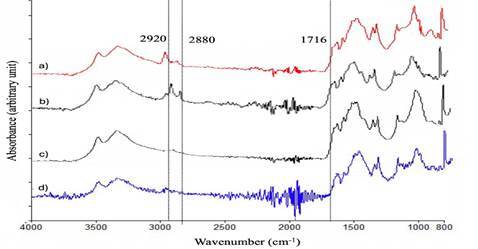
FTIR spectrum of a) glass ionomer cement non-incorporated (control group); b) glass ionomer cement incorporated with 10% chitosan; c) glass ionomer cement incorporated with 10% nanodiamond; and d) glass ionomer cement incorporated with 5% chitosan and 5% nanodiamond. Peaks at 2920 and 2880 cm^−1^ correspond to C-H stretching vibrations from chitosan, while a small peak at 1716 cm^−1^ corresponds to N-C=O stretching, indicating the reaction between amino groups and carboxyl groups.

Four materials were evaluated. In the control group, Fuji II glass ionomer cement (GC Corporation, Tokyo, Japan) received no incorporation. 10%CH and 10%ND groups were characterized by the incorporation of 10% chitosan and 10% nanodiamond into Fuji II glass ionomer cement based on powder weight, respectively. In the 5%CH-5%ND group, Fuji II restorative material powder was incorporated with 5% chitosan and 5% nanodiamond based on powder weight. Chitosan particles, which have a deacetylation degree of 90% and a molecular weight of 375 kDa (Sigma Chemical Co., St. Louis, Missouri, USA), and/or nanodiamond, carbon particles with an average diameter between 2 and 8 nm (Ebersoles, Nürnberg, Germany), were incorporated into the glass ionomer cement powder and mixed through a mechanical mixer for 5 minutes, and subsequently by an ultrasonic mixer for 3 minutes to obtain a homogeneous consistency of the material. The incorporation of chitosan and/or nanodiamond was confirmed by FT-IR (Spectrum Two, PerkinElmer, MA, USA) (Figure 1) ^10^.

Fifty-six glass ionomer cement samples (5 mm-diameter and 2 mm-thick) were made using a silicone matrix (Express XT, 3M ESPE, St. Paul, MN, USA) and divided into four experimental (n=14). The restorative material was handled according to the manufacturer's recommendations and inserted into the silicone matrix using a syringe delivery system to prevent the incorporation of air bubbles. The silicone matrix was covered with a Mylar strip and glass microscope slide to flatten the glass ionomer cement. The samples were manually flattened using #600, #800, and #1200 grit silicon carbide papers (Buehler, Lake Bluff, IL, USA) under water cooling, and polished with #6, #3, #1, and #0.25 diamond pastes (Buehler, Lake Bluff, IL, USA) for a period of 3 minutes for each paste. Then, the samples were cleaned in an ultrasonic unit (Cristófoli, Campo Mourão, PR, Brazil) with deionized water for 5 min to remove residues between each finishing and polishing step, and at the end of the process. Subsequently, the samples were dried with air jets.

### Color Stability

Ten samples (n=10) were submitted to an initial chromatic analysis, using a UV-visible (VIS) spectrophotometer (model UV-2450; Shimadzu, Kyoto, Japan) using the Commission Internationale de l'Eclairage (CIE) L*a*b* system. This consists of two axes, which have right angles and represent the dimension of hue or color (a*: red-green ratio; b*: yellow-blue ratio). The third axis is brightness, represented by the letter L*. This is perpendicular to the a*b* plane. A demarcation was made on the posterior portion of each sample to allow its insertion standardization in the color analysis device. Before each measurement, the spectrophotometer was calibrated with a white barium sulfate background. Five color measurements were taken for each sample under the D65 illuminant, and the values were averaged to obtain the arithmetic mean. The room lighting was dimmed, and the temperature was maintained at 20°C ± 1°C, with controlled relative humidity set at 44% ± 6%.

Color stability was determined by the difference (*Δ*E_00_) between the coordinates obtained from the samples before and after the aging procedure. The color change, *Δ*E_00_, is commonly used to represent a color difference and is calculated using the formula ^13^:

where ΔL´, ΔC´, and ΔH´ indicate the differences in lightness, chroma, and hue, respectively; R_T_ is the rotation function; S_L_, S_C_, and S_H_ are weighting functions. The parametric factors K_L_, K_C_, and K_H_ were considered to be 1. The 50:50% perceptibility level for *Δ*E_00_ was determined at 0.8 (PT = 0.8) while the 50:50% acceptability level was determined at 1.8 (AT = 1.8) ^13^.

### Surface Roughness

Surface roughness was determined, before and after aging in red wine solution, using a profilometer (Surftest SJ 401; Mitutoyo Corp., Tokyo, Japan) (n=10). Previously, the equipment was calibrated using a roughness reference calibrator (R_a_ = 3 µm). The needle tip was positioned in the center of the specimen surface and R_a_ and R_z_ values were measured using a cut-off of 0.8 mm at a speed of 0.1 mm/s. Three readings were taken on each surface at different positions, rotating the specimen 120 degrees, and an arithmetic mean was calculated ^12^.

### Fluorescence Intensity

Fluorescence readings were performed on all samples, before and after aging, using a spectrofluorometer (RF-5301 PC; Shimadzu Corp., Kyoto, Japan) (n=10). The specimens were fixed in the spectrofluorometer with the excitation beam incident at the center of the sample (370 nm), with emission slits of 1.5 nm of aperture. Data obtained were recorded on the spectrofluorometer software in the form of graphs, recording all values of fluorescence intensity that are in the visible light spectrum between 400 nm to 600 nm. The average of fluorescence intensity values between 420 nm to 470 nm wavelength, corresponding to the visible light spectra between violet and blue, was calculated. Three readings were performed for each specimen and an arithmetic mean was calculated.

### Knoop Hardness

The specimens were submitted to a microhardness tester (Micromet 5114; Buehler, Lake Bluff, USA) to verify the microhardness of the glass ionomer cement surface before and after aging (n=10). Five indentations were performed using a load of 25 g for 5 s, and an arithmetic mean was calculated. Knoop hardness values were obtained using the microhardness tester´s software (Buehler OmniMet; Buehler, Lake Bluff, USA). After the initial hardness measurements, the samples underwent repolishing according to the previously described protocol.

### Water Sorption

A 0.01 mg precision digital analytical balance (ATY-224; Shimadzu, São Paulo, SP, Brazil) was used to obtain the initial and final mass of each sample before and after the aging process (n=10). Each specimen was weighed three times, and then an arithmetic mean was calculated. Water sorption (WS) per unit volume (mg/mm^3^) was calculated using the following formula ^14^:

$$WS=\frac{M_{final}-M_{initial}}{V}$$

$$V=\pi \;x\;r^{2}x\;h\;$$

where M_final_ is the mass of the sample after the aging process, M_initial_ is the mass before aging in red wine solution, V is the volume of the sample, π is 3.14, r is the radius of the sample, and h is the height of the specimen.

### Hygroscopic Expansion

For the analysis of hygroscopic expansion, three measurements (extreme right portion, central portion, and extreme left portion of the samples) were performed to measure the thickness of each specimen before and after the aging using a digital caliper (model 500-144B; Mitutoyo Sul América Ltda, SP, Brazil) (n=10). Then an arithmetic mean was calculated. Hygroscopic expansion (expressed as volume change) was measured using the following formula ^14^ ^,^ ^15^:

$$\Delta V\;(\%)\;=\;\&\#091;{\left(H_{final}\;-\;H_{initial}\right)}^{3}\;-\;1\&\#093;\;x\;100$$

where H_final_ is the thickness of the sample after the aging process and H_initial_ is the thickness before aging in red wine solution.

### Aging Process

After the initial readings of color stability, surface roughness, fluorescence intensity, microhardness, water sorption, and hygroscopic expansion, samples from each group were individually immersed in lightproof containers with 5 ml of red wine solution (Valdorella, Malbec, Argentina; pH: 3.5) for a period of 28 days, kept in a laboratory oven (ECB-2; Adamo Products for Laboratory Ltd, Piracicaba, Sao Paulo, Brazil) at 37°C. The containers were sealed to prevent the solution evaporation, which was changed weekly ^12^. After the aging process, the following analyses were conducted: color stability, surface roughness, fluorescence intensity, water sorption, hygroscopic expansion, contact angle, surface free energy, total free energy of interaction, and finally, microhardness.

### Contact Angle, Surface Free Energy, and Total Free Energy of Interaction

The glass ionomer cement surface free energy (γs) and it's nonpolar (γ^LW^: Lifshiz van der Waals) and polar (γ^AB^: acid/base) components were calculated by contact angle measurements on the restorative material using an automatic goniometer (DSA 100S; Krüss, Hamburg, Germany) (n=10). Three specific solutions with established surface energy parameters were used: water (polar), methylene iodide (apolar), and ethylene glycol (polar with acid/base component) ^16^.

0.3 μL of each solution was automatically dropped on three specific regions, previously determined for each solution, using a glass syringe (500 μL) and 0.5 mm needle of caliber. The contact angle was determined by the drop image captured by the software (Drop Shape Analysis DSA4 Software, version 2.0-01; Krüss, Hamburg, Germany) installed in the goniometer, and then measured by the tangent method. Each drop was measured five times for 5 seconds at 20^º^C ± 1°C and relative humidity of 44% ± 6%, and an arithmetic mean was calculated. The parameters, such as Lifshiz van der Waals (γ^LW^, nonpolar component), Lewis acid-base (γ^AB^, polar component), acid component (γ+; receptor component), and base component (γ-; donor component) of surface free energy (mN/m) were calculated to determine the free energy of substrate interaction according to the following equation ^16^:

$$\left(1\;+\;cos\;\theta )\;\gamma_{s}\;=\;-2\;(\sqrt{\gamma s}\;LW\;-\;\sqrt{\gamma_{L}}\;LW)\;+\;(\sqrt{{\gamma_{s}}^{+}}{\gamma_{L}}^{-}\;+\;\sqrt{{\gamma_{s}}^{-}}{\;\gamma_{L}}^{+}\right)$$

The total free energy of interaction (ΔG_sws_^Total^) between the restorative material and water was measured to determine the hydrophilicity/hydrophobicity of the glass ionomer cement surface, according to the following formula ^16^:

$${∆G}_{sws}Total\;=\;-2\;{\left(\sqrt{\gamma_{s}}\;LW\;-\;\sqrt{\gamma_{w}}\;LW\right)}^{2}\;-\;4\;\left(\sqrt{{\gamma_{s}}^{+}}+{\gamma_{s}s}^{-}-\;+\;\sqrt{{\gamma_{w}}^{+}}+{\gamma_{w}}^{-}\;-\;\sqrt{{\gamma_{s}}^{-}}+{\gamma_{w}}^{-}\;-\;\sqrt{{\gamma_{s}}^{-}}\;{\gamma_{w}}^{+}\right)$$

where ΔG_sws_^Total^ > 0 characterizes the surface as a hydrophilic surface and ΔG_sws_^Total^ < 0 as a hydrophobic surface.

### Scanning Electron Microscope (SEM) and Energy Dispersive X-Ray Spectroscopy (EDS)

Two unaged and two aged samples from each experimental group were fixed in metallic stubs and sputter-coated with gold (Baltec SCD 050; Balzers, Liechtenstein, Austria) (n=4). Both analyses (SEM and EDS) were performed to qualitatively evaluate the morphology and chemical composition of the experimental groups before and after aging. Before analysis, the specimens underwent cleaning in an ultrasonic unit (Cristofoli, Campo Mourão, PR, Brazil) with distilled water for 8 minutes to eliminate contaminants or residues. Subsequently, to ensure the complete removal of any remaining moisture, the samples were dried in a drying oven at 100 ºC for 5 minutes. Glass ionomer cement morphology micrographs were obtained using scanning electron microscopy under ×500 and ×2000 magnification, and the substrate composition was evaluated qualitatively by energy-dispersive X-ray spectroscopy coupled to the SEM equipment (JSM5600LV; JEOL, Tokyo, Japan). Data on carbon (C), oxygen (O), fluorine (F), sodium (Na), gold (Au), aluminum (Al), silicon (Si), phosphorus (P), chlorine (Cl), potassium (K), calcium (Ca), titanium (Ti), strontium (Sr), barium (Ba), and magnesium (Mg) were collected from the SEM-EDS analysis under ×500 magnification ^16^.

### Statistical analysis

Data were submitted to normality (Shapiro-Wilk; Bioestat 2.0 Program) and homogeneity tests (Bartlett; Bioestat 2.0 Program). Color stability, water sorption, hygroscopic expansion, contact angle, surface free energy, and total free energy of interaction were submitted to analysis of variance (ANOVA; 5.0 Statview Program; Version 5.0.1). Surface roughness, fluorescence intensity, and microhardness were analyzed using 2-way repeated measures ANOVA (5.0 Statview Program; Version 5.0.1). The Tukey protected least significant difference test (α=.05) was also performed for all the analyses mentioned above.

## Results

### Color Stability

**Figure 2:**
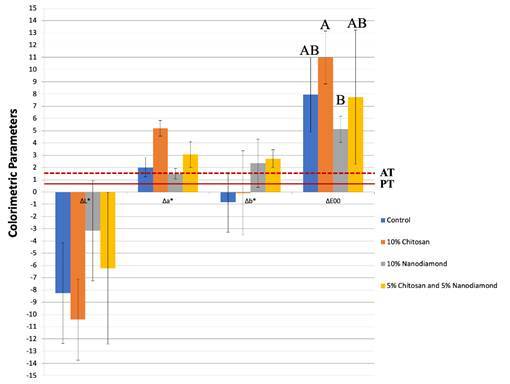
Mean ± standard deviation values of colorimetric parameters (*Δ*L*, *Δ*a*, and *Δ*b*) as a function of glass ionomer cement experimental groups. The continuous line at 0.81 *Δ*E_00_ units and the dashed line at 1.77 *Δ*E_00_ units represent the perceptibility (PT) and acceptability (AT) thresholds for the *Δ*E_00_ parameter, respectively. Different letters indicate statistically significant differences in the *Δ*E_00_ data (P<.05).

The colorimetric parameters and color stability values are listed in Figure 2, respectively. 10% chitosan-incorporated glass ionomer cement group showed higher negative *Δ*L* and positive *Δ*a* values (L* negative - darker samples; a* positive - towards red), while 5% chitosan- and 5% nanodiamond-incorporated group presented higher positive *Δ*b* values (b* positive - towards yellow) (Figure 2). There was a significant difference in the comparison of color stability among the experimental groups of glass ionomer cement (P<.05). The incorporation of 10% chitosan into the restorative material promoted higher chromatic alteration values when compared to the 10% nanodiamond-incorporated glass ionomer cement group (P=.0037). No differences were comparing the group of no incorporated glass ionomer cement (control) about the other experimental groups (P>.05). Figure 2 illustrates that all experimental groups showed *ΔE*_*00*_ values higher than the perceptibility (PT = 0.81) and the acceptability (AT = 1.77) thresholds.

### Surface Roughness

The surface roughness parameters ​​(R_a_ and R_z_) values are illustrated in Table 1. There were no differences in R_a_ and R_z_ values among the glass ionomer cement groups before aging (P>.05). However, after the aging process in red wine solution, the 10% chitosan-incorporated glass ionomer cement group presented higher R_a_ (P=.0083) and R_z_ (P=.0325) values about the 10% nanodiamond-incorporated glass ionomer cement group (Table 1). There were no differences in surface roughness parameters (R_a_ and R_z_) comparing the group of no incorporated glass ionomer cement (control) to the other experimental groups (P>.05). Regarding the aging process, it can be noted that the red wine solution promoted greater changes for both surface roughness parameters (R_a_ and R_z_) of all glass ionomer cement groups when compared to the values ​​before aging (P<.05) (Table 1).

**Table 1:** Mean ± standard deviation values of surface roughness R_a_ (μm) and R_z_ (μm) as a function of glass ionomer cement experimental groups before and after aging in red wine solution.

Groups	Control	10% Chitosan	10% Nanodiamond	5% Chitosan and 5% Nanodiamond
R_a_				
Before Aging	0.78 ± 0.29 B a	0.80 ± 0.16 B a	0.62 ± 0.14 B a	0.74 ± 0.09 B a
After Aging	3.35 ± 1.69 A ab	4.52 ± 1.94 A a	2.03 ± 1.15 A b	3.51 ± 1.10 A ab
R_z_				
Before Aging	6.19 ± 2.61 B a	5.02 ± 1.70 B a	4.34 ± 1.94 B a	5.54 ± 1.22 B a
After Aging	19.82 ± 10.22 A ab	25.98 ± 9.02 A a	13.48 ± 6.10 A b	22.52 ± 10.94 A ab

Different letters, uppercase in column and lowercase in row, indicate statistically significant differences for each parameter (P<.05).

### Fluorescence Intensity

The fluorescence intensity values are described in Table 2. 10% chitosan-incorporated, 5% chitosan- and 5% nanodiamond-incorporated glass ionomer cement groups promoted higher fluorescence intensity values compared to the control group before aging (P=.0030). However, after aging, the opposite trend occurred once the control group showed higher fluorescence intensity values compared to 10% chitosan-incorporated and 5% chitosan- and 5% nanodiamond-incorporated glass ionomer cement groups (P=.0015) (Table 2). No differences were comparing the 10% nanodiamond-incorporated glass ionomer cement and control groups before and after the aging process (P>.05). Red wine solution promoted lower fluorescence intensity values ​​for all experimental groups when compared to pre-cycling values ​​(P<.05), except for the control group (P>.05) (Table 2).

**Table 2:** Mean ± standard deviation values of fluorescence intensity as a function of glass ionomer cement experimental groups before and after aging in red wine solution.

Groups	Control	10% Chitosan	10% Nanodiamond	5% Chitosan and 5% Nanodiamond
Before Aging	40.03 ± 14.59 A b	112.60 ± 57.00 A a	86.21 ± 34.92 A ab	99.21 ± 49.31 A a
After Aging	66.51 ± 30.75 A a	23.88 ± 13.86 B b	48.84 ± 33.15 B ab	27.57 ± 16.61 B b

Different letters, uppercase in column and lowercase in row, indicate statistically significant differences (P<.05).

### Knoop Hardness

The Knoop microhardness values are shown in Table 3. The 10% nanodiamond-incorporated glass ionomer cement group showed higher hardness values before aging when compared to the other experimental groups (P=.0005). However, after the aging process, there were no significant differences among all glass ionomer cement groups (P=.4529). The aging process promoted a significant decrease in Knoop microhardness values for all experimental groups (P<.0001) (Table 3).

**Table 3:** Mean ± standard deviation values of Knoop microhardness as a function of glass ionomer cement experimental groups before and after aging in red wine solution.

Groups	Control	10% Chitosan	10% Nanodiamond	5% Chitosan and 5% Nanodiamond
Before Aging	88.63 ± 26.49 A b	82.63 ± 18.79 A b	123.03 ± 28.36 A a	82.92 ± 12.23 A b
After Aging	35.84 ± 12.30 B a	32.37 ± 11.08 B a	33.19 ± 9.59 B a	39.09 ± 6.67 B a

Different letters, uppercase in column and lowercase in row, indicate statistically significant differences (P<.05).

### Water Sorption

The water sorption values are shown in Table 4. 10% chitosan-incorporated glass ionomer cement group presented higher water sorption values in relation to the restorative material incorporated with 5% of chitosan and 5% of nanodiamond (P=.0196). There were no differences on water sorption comparing the group of no incorporated glass ionomer cement (control) in relation to the other experimental groups (P>.05) (Table 4).

**Table 4:** Mean ± standard deviation values of water sorption (mg/mm^3^) as a function of glass ionomer cement experimental groups.

Groups	Control	10% Chitosan	10% Nanodiamond	5% Chitosan and 5% Nanodiamond
	126.61 ± 16.13 AB	133.13 ± 18.68 A	115.53 ± 11.56 AB	114.67 ± 10.82 B

Different letters indicate statistically significant differences (P<.05).

### Hygroscopic Expansion

The hygroscopic expansion values are listed in Table 5. It can be noted that all experimental groups showed a volumetric decrease after storage in red wine solution. There were no significant differences among the glass ionomer cement groups evaluated (P=.0573) (Table 5).

**Table 5:** Mean ± standard deviation values of hygroscopic expansion (%) as a function of glass ionomer cement experimental groups.

Groups	Control	10% Chitosan	10% Nanodiamond	5% Chitosan and 5% Nanodiamond
	-2.69 ± 1.57 A	-1.41 ± 1.51 A	-3.18 ± 3.46 A	-2.38 ± 2.90 A

Different letters indicate statistically significant differences (P<.05).

### Contact Angle, Surface Free Energy, and Total Free Energy of Interaction

The contact angle, surface free energy, and total free energy of interaction values are described in Table 6. There were no differences among the experimental groups for contact angle (P=.1067) and total free energy of interaction analyses (P=.1397). However, for surface free energy analysis, the 10% nanodiamond-incorporated glass ionomer cement group promoted higher values compared to the 10% chitosan-incorporated material and control groups (P=.0301) (Table 6).

**Table 6:** Mean ± standard deviation values of contact angle (º), surface free energy γs (mN/m), and total free energy of interaction - Delta G (mJ/m^2^) as a function of glass ionomer cement experimental groups.

Groups /Analyses	Control	10% Chitosan	10% Nanodiamond	5% Chitosan and 5% Nanodiamond
Contact Angle	73.60 ± 7.84 A	55.98 ± 18.17 A	46.31 ± 15.23 A	60.12 ± 21.27 A
Surface Free Energy	31.98 ± 2.32 B	32.22 ± 3.70 B	41.68 ± 8.92 A	38.89 ± 3.25 AB
Delta G	-41.13 ± 39.40 A	-69.82 ± 13.21 A	-46.86 ± 60.54 A	-93.68 ± 9.34 A

Different letters in a row indicate statistically significant differences.

### Scanning Electron Microscope (SEM) and Energy Dispersive X-Ray Spectroscopy (EDS)

**Figure 3:**
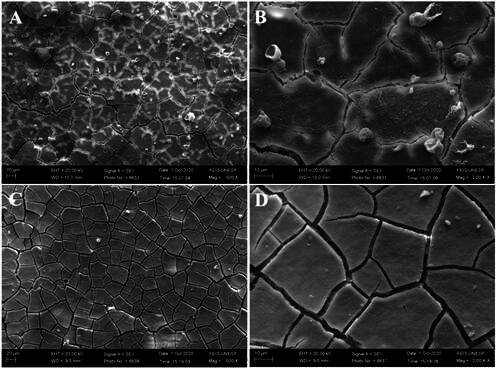
Scanning electron micrographs of glass ionomer cement non-incorporated (original magnification ×500 and ×2000). A, B - Before aging. C, D - After aging.

**Figure 4:**
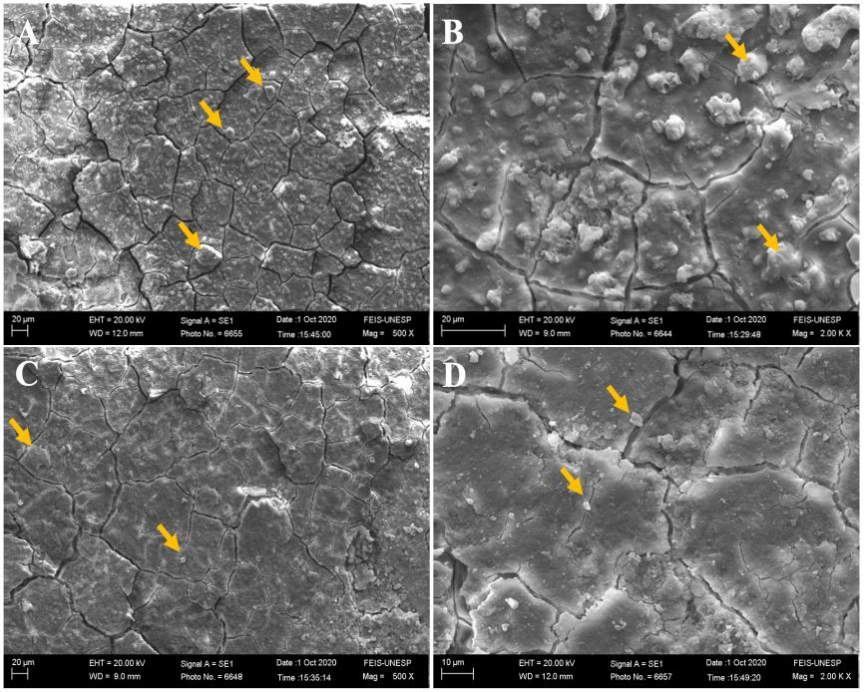
Scanning electron micrographs of glass ionomer cement incorporated with 10% chitosan (original magnification 500 and ×2000). A, B - Before aging. C, D - After aging. Yellow arrows represent the chitosan particles.

**Figure 5.**
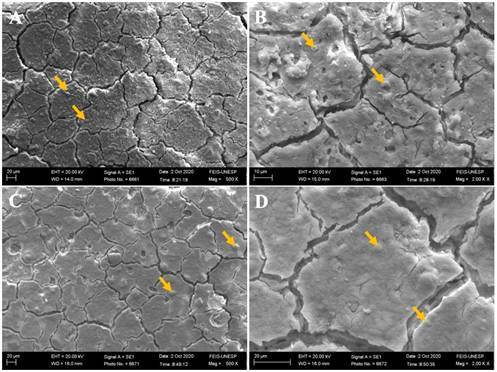
Scanning electron micrographs of glass ionomer cement incorporated with 10% nanodiamond (original magnification ×500 and ×2000). A, B - Before aging. C, D - After aging. Yellow arrows represent the nanodiamond particles

**Figure 6:**
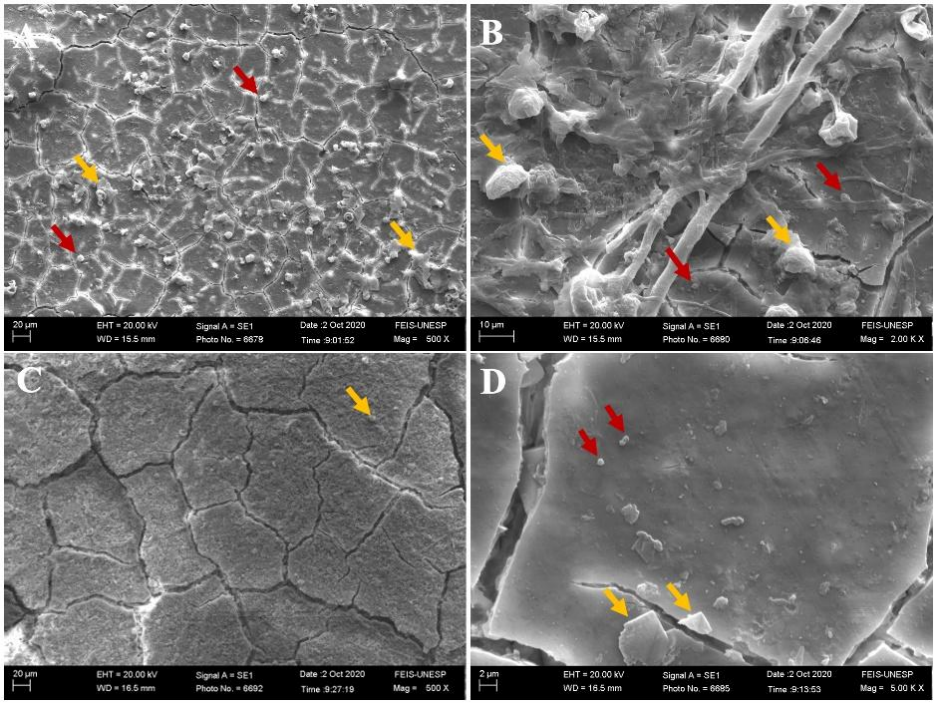
Scanning electron micrographs of glass ionomer cement incorporated with 5% chitosan and 5% nanodiamond (original magnification ×500 and ×2000). A, B - Before aging. C, D - After aging. Yellow arrows represent the chitosan particles and red arrows represent the nanodiamond particles.

**Figure 7:**
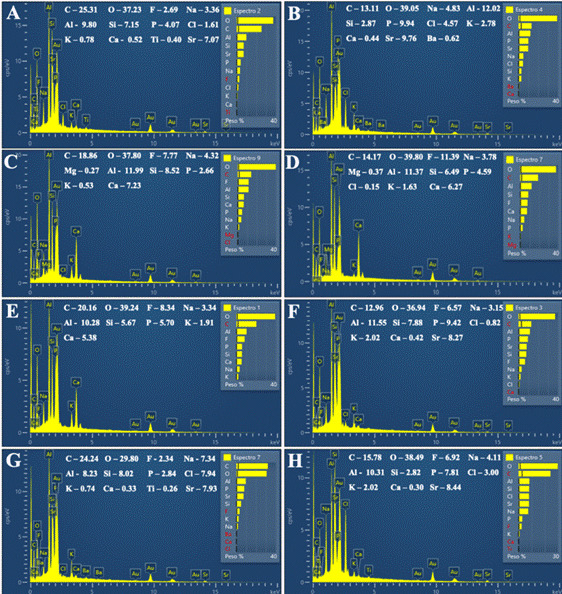
Energy dispersive x-ray spectra of the chemical elements (percentage) of glass ionomer cement according to the chitosan and nanodiamond incorporation before and after aging. A - non-incorporated glass ionomer cement before aging. B - non-incorporated glass ionomer cement after aging. C - 10% chitosan-incorporated glass ionomer cement before aging. D - 10% chitosan-incorporated glass ionomer cement after aging. E - 10% nanodiamond-incorporated glass ionomer cement before aging. F - 10% nanodiamond-incorporated glass ionomer cement after aging. G - 5% chitosan- 5% nanodiamond-incorporated glass ionomer cement before aging. H - 5% chitosan- 5% nanodiamond-incorporated glass ionomer cement after aging.

Scanning electron micrographs and energy-dispersive X-ray spectra before and after the aging process are illustrated in Figures 3 to 7. The scanning electron micrographs demonstrate a rough and friable surface of the restorative material regardless of the incorporation or not of chitosan and nanodiamond, and regardless of its concentration of incorporation (Figs. 3, 4, 5, 6). It can be observed the presence of chitosan particles on the surface of the 10% chitosan-incorporated glass ionomer cement group (Figure 4), as well as the presence of nanodiamond particles on the surface of the 10% nanodiamond-incorporated glass ionomer cement group (Figure 5). In the 5% chitosan- and 5% nanodiamond-incorporated glass ionomer cement group, it can be observed the simultaneous presence of chitosan and nanodiamond particles on the surface of the restorative material (Figure 6). The energy dispersive X-ray spectra indicated a reduction in the percentage concentration of the carbon element (C) across all experimental groups following aging in red wine solution (Figure 7). This chemical element is the main component of chitosan- and nanodiamond-modified ionomer cement.

## Discussion

The chitosan and nanodiamond incorporation into glass ionomer cement influenced the optical, mechanical, and surface properties of the restorative material; thus, the first null hypothesis was rejected. Analyses of the influence of the aging process in red wine solution on surface roughness, fluorescence intensity, and microhardness led to a rejection of the second null hypothesis.

Glass ionomer cement is one of the main bioactive restorative materials used in atraumatic restorative treatment, since it chemically adheres satisfactorily to the tooth structure, allowing the release of fluoride, reducing bacterial proliferation, and favoring the remineralization process of the dental structure ^1^ ^,^ ^2^ ^,^ ^3^ ^,^ ^4^. According to Mishra et al., glass ionomer cements release fluoride ions approximately 10 ppm during the 48 hours after its application into the oral cavity ^17^. However, this release can be considered inefficient for an effective and desirable antibacterial effect. Thus, authors have studied the incorporation of antibacterial agents into glass ionomer cement composition, such as chlorhexidine digluconate and chitosan ^17^.

Nishanthine et al. have asserted that the incorporation of chitosan nanoparticles enhances the fluoride release characteristics of glass-ionomer cements. This is because chitosan nanoparticles induce structural modifications in the cement matrix, facilitating the more efficient diffusion of fluoride ions ^5^. In a related study, Elshenawy et al. evaluated the impact of incorporating quaternized chitosan-coated mesoporous silica nanoparticles into conventional glass ionomer cement ^8^. Their research encompassed an assessment of mechanical properties, antimicrobial activity, and fluoride release following an aging process ^8^. The findings from their study indicated that chitosan nanoparticles hold significant promise as fillers in dental materials. They contribute to strengthening the material, increasing fluoride release, enhancing physico-mechanical attributes, and providing antibacterial capabilities against *Streptococcus mutans* ^8^. These advancements represent valuable contributions to the enhancement of dental materials.

Chitosan is a cationic polysaccharide produced through the deacetylation process of chitin, and this polysaccharide is widely used in biomedical applications due to its high biocompatibility and antimicrobial properties ^1^ ^,^ ^2^ ^,^ ^3^ ^,^ ^4^. Chitin is a yellowish powder with a crystalline or amorphous structure, highly hydrophobic and insoluble in water ^1^ ^,^ ^2^ ^,^ ^3^ ^,^ ^4^. These characteristics may have corroborated that the glass ionomer cement groups incorporated with 10% chitosan, 5% chitosan, and 5% nanodiamond showed higher contact angle compared to 10% nanodiamond-incorporated restorative material and higher negative Delta G values compared to control and 10% nanodiamond groups, despite the non-statistical difference among the groups (Table 6).

Chitosan presents a powder with heterogeneous granulation, darker than chitin, slightly yellowish, and a drier texture ^1^ ^,^ ^2^ ^,^ ^3^ ^,^ ^4^. These chromatic characteristics may have influenced the final values of color stability analysis since 10% chitosan-incorporated glass ionomer cement presented higher delta negative values of the colorimetric parameters for the "L" and higher values for the "a" axis. As a result, this experimental group exhibited higher sorption values (Table 4) which directly contributed to higher *Δ*E_00_ values, exceeding both the perceptibility (PT = 0.81) and the acceptability (AT = 1.77) thresholds, despite the lack of statistically significant differences when compared to the control group (Fig. 2). Furthermore, chitosan oligomer is composed of β-^1^ ^,^ ^4^-2-amido-2-deoxy-D-glucan and β- ^1^ ^,^ ^4^-2-acetoamido-2-deoxy-D-glucan (acetylglucosamine) that present specific structural and fluorescent characteristics, and the reaction intensity between the amino group presented in chitosan structure and carbon dioxide in air can enhance the fluorescence intensity ^18^ ^,^ ^19^. Enamel and dentin are both fluorescent tissues, yet enamel typically exhibits relatively weak fluorescence due to its low organic content. Teeth commonly emit a bluish-white hue when exposed to ultraviolet (UV) light. Specifically, the fluorescence spectrum of natural enamel reveals maximum luminescence peaks around 450 nm, whereas dentin demonstrates peaks at approximately 440 nm ^20^. These intrinsic characteristics of chitosan could explain the higher fluorescence intensity by chitosan-incorporated glass ionomer cement groups before aging compared to the control group (Table 2), which is consistent with the fluorescence patterns exhibited by tooth substrates.

Nanodiamond particles have been widely investigated due to their incorporation into polymeric matrices of restorative materials to improve their mechanical properties ^9^ ^,^ ^11^. The reason nanodiamond particles are inserted into dental material composition is due to their inertia ^9^. However, its surface is still reactive, making it a biocompatible material ^9^. This statement can be noted by the results obtained in the present study, where the 10% nanodiamond-incorporated glass ionomer cement group presented higher surface free energy values compared to the control and 10% chitosan groups (Table 6). The increase of surface free energy may have promoted the nanodiamond particle aggregation, corroborating lower surface roughness values (R_a_ and R_z_) before and after the aging process of the 10% nanodiamond-incorporated glass ionomer cement group compared to other groups, despite the non-statistical difference (Table 1) ^9^ ^,^ ^21^.

The surface roughness of conventional glass ionomer cement is directly related to some factors, such as the size and shape of the glass particles, adhesion between the particles and the matrix, inherent resistance to cement constituents, and the setting reaction of each material type ^21^. In the field of dentistry, the correlation between surface roughness and bacterial adhesion to dental restorative materials is extremely important. Generally, rough surfaces are more likely to accumulate more bacterial biofilm ^7^. Bollen et al. have established a surface roughness threshold value of 0.2 µm R_a_, and an increase above this threshold would increase bacterial adhesion ^7^. Nanodiamonds are carbon nanoparticles with an octahedral structure similar to a diamond with a diameter of approximately 2 to 8 nm ^9^. Thus, it can be speculated that the lower surface roughness (R_a_ and R_z_) values for the 10% nanodiamond-incorporated glass ionomer cement group are because the nanodiamond particles have filled the intermediate spaces between the constituent particles of the glass ionomer material, yielding lower surface roughness (Table 1). The surface roughness parameters R_a_ and R_z_ were both adopted to provide a comprehensive assessment of the material's surface characteristics. R_a_ offers a general overview of the surface texture by calculating the average deviation of peaks and valleys, while R_z_ focuses on the difference between the highest peaks and deepest valleys, capturing more detailed surface irregularities. The combination of these two parameters allows for a more robust evaluation of the effects of material modifications and features of surface topography.

As a type of carbon-based nanomaterial, nanodiamond particles are currently considered a promising nano-additive for biomedical applications due to their nanometer-scale particle size, in addition to their satisfactory biocompatibility and mechanical properties ^9^. Cao et al. evaluated the incorporation of nanodiamond particles functionalized with hydrophilic cationic copolymer into composite resins composition improving the mechanical properties, such as hardness, strength, and flexural modulus, in addition to improving the antibacterial activity of the composite ^9^. The incorporation of 10% nanodiamond into the glass ionomer cement promoted higher initial hardness values to the restorative material of the other groups (Table 3), corroborating the results found by Cao et al ^9^.

The implementation of an aggressive and prolonged exposure protocol involving immersion of the specimens in red wine solution for 28 days significantly impacted the optical and mechanical properties of the glass ionomer cement, regardless of the concentration of chitosan and/or nanodiamond incorporated, as well as in the non-incorporated counterpart (control group) (Tables 1 to 3). It is speculated that the reduced pH of the red wine solution may have caused the softening of the polysalt matrix of the glass ionomer material, being this material is relatively soluble in acidic solutions compared to neutral solutions ^22^ ^,^ ^23^. It is important to emphasize that the acid pH used in the present study may not represent the clinical condition of the oral cavity due to the absence of the solution dilution by saliva, brushing of the teeth, and the period of wine solution contact with the teeth and/or restorations. The decrease in the mechanical properties values (Tables 1 and 3) may be directly related to the effect of red wine solution on the restorative material, speculating that the ethyl solution promoted a disintegration and hydrolytic degradation in the polysalt matrix and/or in the filler loads ^12^ ^,^ ^23^, exacerbating the formation of micro-cracks and fissures in the glass ionomer material as can be seen in the scanning electron micrographs (Figs. 3 to 6).

The energy dispersive x-ray spectra showed that aging in red wine solution was able to decrease the percentage concentration of the carbon element (C) in the incorporated groups, as well as in the non-incorporated counterpart (control group) (Fig. 7). This element is the main constituent of chitosan (C_6_H_11_O_4_N)_n_ and nanodiamond (C)_n_ compounds and its decrease could suggest its detachment from the organic matrix due to its high solubility to the aqueous medium corroborating with the data obtained by EDS analysis (Fig. 7). These findings may be attributed to the molecular weight of chitosan and the method used to incorporate both components into the glass ionomer cement. However, additional research is needed to validate the correlation of these factors conclusively.

Furthermore, the ions and chemical elements loss from the restorative material promotes the voids and gaps formation that can be observed in SEM images (Figs. 3 - 6), allowing the filling of spaces by the solution resulting in water sorption and the material mass increase ^14^ ^,^ ^15^. This statement can be corroborated by the increased values of water sorption found in the present study for all experimental groups evaluated (Table 4). However, it is important to consider that the restorative material surface to be exposed to fluids in a clinical situation may be smaller than in a laboratory condition, which probably will reduce the solubility of the glass ionomer cement ^15^.

An interesting finding of the present study is that the experimental groups showed negative values of hygroscopic expansion (Table 5), and shrinkage after immersion in wine solution, despite the water sorption values being positive (Table 4). According to Mustafa et al ^14^. and Sidhu et al. ^24^, this result can be explained by the self-healing effect of the material, which implies that the cracks and internal fissures (Figs. 3 - 6) that develop in glass ionomer material when dehydrated tend to be repaired by rehydration ^14^ ^,^ ^24^. Thus, the shrinkage of the glass ionomer groups may be related to the healing effect of the solution, which tends to reduce internal cracks, reducing the volume of the restoration ^14^ ^) and^ corroborating with the hygroscopic expansion values of the present study (Table 5).

Although the results of the present study show that the incorporation of 5% chitosan and 5% nanodiamond into glass ionomer cement did not generally promote a significant difference compared to the control group when evaluating the optical, mechanical, and surface properties (Tables 1 to 6), it is important to emphasize the effectiveness of biocompatibility, biodegradability, adhesive properties to the dental substrate, anti-inflammatory and antibacterial properties, prevention of demineralization of dental tissues, and inhibition of bacterial plaque accumulation of these components already scientifically proven ^25^. Thus, the association of 5 % chitosan with 5% nanodiamond becomes a satisfactory concentration in the incorporation into glass ionomer cement since they contain antibacterial activity, and the association of both components did not promote damage to optical, mechanical, and surface properties of the restorative material.

Understanding the compound behavior used in dental composite formulation becomes essential in the improvement and consequently in the longevity of these materials in the oral cavity. Thus, future studies are necessary to complement the discussions around the improvement of the mechanical and biological properties of glass ionomer cements, as well as to evaluate their performance over time. Some limiting factors of this study must be taken into account, such as the use of only one type of glass ionomer cement, the assessment of only two concentrations of chitosan and nanodiamond, and because this study is characterized as an *in vitro* research, the transfer of laboratory-level results to clinical conditions should be performed with caution, since *in vitro* studies cannot reliably simulate the condition of the oral cavity, such as the interference of occlusal loads, temperature, microorganism, and enzymes. Furthermore, the aging protocol adopted in the present study was more aggressive, with specimens exposed for a longer duration to a wine solution, which would likely result in greater degradation of the samples. Further investigations are required to determine the influence of chitosan and nanodiamond incorporation into glass ionomer cements on permeability, fluoride-releasing, marginal adaptation, and other physical-mechanical properties aimed at improving the features and clinical longevity of the restorative material.

## Conclusion

Based on the methodology and findings of this *in vitro* study, it can be concluded that 10% chitosan and 10% nanodiamond incorporation influenced the color stability, surface roughness, fluorescence intensity, microhardness, water sorption, and surface free energy of the glass ionomer cement. The incorporation of 5% chitosan and 5% nanodiamond is a promising alternative for maintaining the surface, optical, and mechanical properties of glass ionomer cement, showing a performance comparable to the control group. Glass ionomer cement properties were altered by the aging on red wine solution.
